# Chicago Medical Society, Meeting of September 21st

**Published:** 1885-10

**Authors:** 


CHICAGO MEDICAL SOCIETY.

*Stated meeting, September 21, 1885.* The PRESIDENT, C. T. PARKES, M. D., in the chair.

"*Special versus General Study in Medicine*" was the title of an essay by Dr. Charles F. Sinclair. The following is an abstract: During the seventeenth and eighteenth centuries there existed several schools of medicine founded upon medical investigations stimulated by the discovery of the circulation of the blood by Harvey. These schools were founded upon theories. One school explained the processes of life as due to mechanical laws; another, as due to chemical laws; still another, as due to molecular movements; and finally, schools were founded which respectively promulgated the doctrines that life was due to an ether or a spirit existing in the grosser material of the body. Thus we see that medical thought and investigation began to divide. This subdivision in thought led to separate investigations, and specialism was the result. This tendency, which is becoming more marked each day, may be called, perhaps, the distinguishing characteristic of recent medical study. The more clearly defined and generally recognized specialties to-day are surgery, diseases of the eye, diseases of the ear, diseases of women, diseases of children, diseases of the skin, diseases of the nervous system, diseases of the genito-urinary tract, including syphilis, diseases of the throat, heart and lungs, obstetrics and forensic medicine. The rapidity with which the tendency of special study is being developed is seen in the fact that five years ago one of our oldest medical schools gave special notice of only two of the special subdivisions I have mentioned. However, within three years this school has added a fourth year for the study of the branches mentioned. But it is obected that the student of a specialty should become practically conversant with the details of so-called general medicine. But it is a disastrous step for the young practitioner to try to cultivate a special practice from a general, if the old adage, "Once a general practitioner, always a general practitioner," is true. On the other hand, the specialist gains a footing more readily by confining himself to his specialty. It is further objected that the specialist is apt to forget that there are other important organs in the body beside the one to which he has given his attention. But this objection is puerile, when it is seen that the successful specialist must of necessity study the relations the different organs of the body hold to each other, and remember that any departure from the normal in one organ may be the result of disease in a more remote organ. The medical profession should look with favor upon this subdivision of medical work, because we see such vast accumulations of material in all the various departments of medicine that few would care to undertake, or have the ability, to even peruse our medical literature, much less master all the knowledge collected. In this day of rapid interchange of thought none can be found who can keep abreast of all the discoveries in the various departments of medicine. Another reason for the existence of the specialist is the fact that very few men can secure the expensive instruments necessary to be used in the treatment of the various diseases, nor are there many physicians who could skilfully use them were they so fortunate as to possess them. Many cases cannot be successfully treated without these instruments. In our city, specialties should receive further development, because as they are clearly defined and faithfully adhered to in practice, proportionately does the city rank as a medical center.

DR. F. M. WELLER opened the discussion by asking why the subdivision of the practice of medicine into the special treatment of diseases of the various organs should not be carried further into specialties for various specific diseases? Let us have physicians whose respective specialties shall be catarrh, ague, diphtheria, etc.

DR. W. F. COLEMAN said that while he agreed with the author in the main points in his paper, he thought it should rest with each individual whether he shall confine himself to special practice or emphasize it in general practice. It is advantageous to specialists that they should confine themselves to their chosen fields. But we should not judge of the benefits to be derived from specialties by the individual success of each practitioner, but by the extent each practitioner enriched our literature by the record of his investigations. It is thus that the eye and ear specialist, the specialist in throat and lung diseases, the laryngologist and others have done most to advance medicine.

"*The Treatment of Syphilis*" was the title of a paper read by Dr. L. T. Potter. He said that the treatment of syphilis must be threefold—hygienic, tonic and specific. By the latter is meant the administration of mercurial and iodine preparations. The profession seems to be greatly divided in opinion in regard to the methods of using these remedies and the length of time they should be employed. In scanning the literature on this subject the reporter was surprised to find that those high in authority differed as to these points. The reporter advanced two propositions: first, that neither the iodine preparations alone, nor mercury alone, can always be relied upon as effective in the treatment of syphilis, but that *both* are necessary to eradicate the disease; second, that the duration of treatment must be at least two years, faithfully carried out, no matter how mild an attack. In support of his first proposition the reporter quoted Bartholow, Ringer, Jonathan Hutchinson, Keyes, Bumstead and Taylor as saying that mercury should be given in the primary and secondary stages of syphilis, and iodide of potassium in the tertiary stage. They all agree that both must be used to effect what is called a cure. In support of his second proposition, he quotes Van Buren and Keyes Fournier, Bumstead and Taylor, as insisting upon the treatment extending over a course of two years or more. Diday says the minimum time for treatment is twenty-two months. The two-year course of treatment does not mean the continual administration of mercury or iodine, but at intervals the remedy may be discontinued for a short time, if it seems to have a debilitating effect on the patient. In the light of such unanimity of opinion of eminent authorities upon this question of duration of treatment, it is surprising that intelligent physicians will positively assure their patients that they are *cured* of syphilis at the expiration of a course of treatment lasting from four to six months!! A physician who does so is certainly criminally negligent, and is a misanthrope of the worst type. Then it becomes all to impress upon patients the importance of carrying on the treatment for at least two years.

PROFESSOR E. L. HOLMES commenced the discussion by saying he considered the paper very valuable, because the author lays so much stress upon the importance of thorough and long-continued treatment. One of the most important lessons he had ever received was to treat syphilis according to the plan the gentleman has advocated. Many years ago he had been taught this lesson by sad experience. It had been his lot to see many patients suffering from specific diseases of the eye long after they had been discharged by their physicians as cured. He could not understand how any physician can believe it possible to cure syphilis without carrying out the treatment a long time. In many years' practice in this city he cannot remember of having seen but three primary syphilitic lesions, and these all occurred on some portion of the eye. He saw one man who had on the upper eyelid a sore which resembled and was treated as a burn, until its course decided it to be a chancre. The man would not tell how he obtained it, but it readily disappeared under specific treatment. A great many diseases of the eye occur thus, and the physician is unable to find out how they arise. Many years ago it was taught that iodide of potash, if given in large doses, would effect a sure and speedy cure. But Professor Holmes thought the treatment by large doses of iodide dangerous, as it ameliorates the symptoms so quickly as to cause the physician and patient to abandon the remedy too soon. In this country we do not have so good an opportunity to study syphilis as the Europeans. In Vienna and Prague, where the people live and do not pass from the observation of physicians, their statistics are more valuable and reliable than ours can be. He thought it best to give the patient all the mercury he can bear in the primary and secondary stages. Rub it in the skin and give it internally. Follow this up eighteen months or two years, and then give iodide of potassium later. Every three or four months give a course of treatment for years after. You will have no trouble impressing upon intelligent people the importance of long-continued treatment.

DR. R. TILLEY said he wished to refer to one point not touched in the paper, and that is, patients treated for syphilis are often told by their physicians of the importance of long-continued treatment, but they will not heed these warnings, and do not return. This fact will excuse the physician many times, as it is not in his power to carry out the treatment when he wishes; and thus physicians should often be relieved of the blame of not having treated their patients long enough. He did not think any intelligent physician would advocate treatment under two years, and he believed Keyes, in his last edition, extended the time of treatment to four years. Dr. Tilley was of the opinion that we cannot do without mercury, and yet some in high places teach this doctrine. Those who try to treat syphilis without mercury are certainly responsible for later developments.

DR. J. ZEISLER thought the present treatment of syphilis is not scientific, and that there had been little advance in this direction in the last century. Cases are known in which, after seven years' treatment, symptoms of the disease returned. Take the case of Prof. Zeissl, who died last year. He was infected while opening a bubo several years ago. He certainly knew how to treat himself, and yet he died of cerebral syphilis. This does not look as though the treatment of syphilis is yet founded on a scientific basis. If the discovery of the bacillus of syphilis proves to be correct, it may prove the means of enabling us to treat syphilis scientifically.

PROFESSOR G. C. PAOLI said he is by nature a cosmopolitan, and always selects the best from the writings of all nations. A great many books have been written on this subject, among which Ricord's stand first. Ricord was a man of great talent, experience and powers of observation. He had unexcelled opportunities for study in the Paris Hospital when he was the chief physician. In regard to treatment, all agree that mercury must be administered for a long time. There are syphilitic cases in which mercury is contraindicated, namely, phthisical patients and in albuminuria, unless we believe syphilis is the cause of the albuminuria.

DR. R. TILLEY referred to one point introduced by Dr Zeisler, who referred to Professor Zeissl as having died of cerebral syphilis, claiming that no one would doubt Prof. Zeissl's ability to cure syphilis. But the question is not whether Prof. Zeissl knew how to treat syphilis, but how did he treat himself? Cooper, in his book on syphilis, if he (Dr. Tilley) were not greatly mistaken, cites Prof. Zeissl as a type of those who used mercury sparingly. If that is so, and Zeissl used it only sparingly on himself, then the death of Zeissl from cerebral syphilis is a very important lesson, and bears materially on the subject under discussion.

DR. J. ZEISLER said he knew that in the case of Prof. Zeissl mercurial inunctions were made, but to what extent he was unable to say.

DR. L. T. POTTER closed the discussion by expressing himself as gratified at the amount of discussion which had been aroused; however, he was surprised at the statement that there had been little or no advancement in the treatment of syphilis. In this day of elegant pharmaceutical preparations and easy administratisn of mercurials and iodides, he thought there had been a great advancement, for pharmacy and chemistry have stepped in and given us preparations we did not use many years ago. Mercury should not be given when it is producing a debilitating effect.

The President, PROFESSOR C. T. PARKES, presented to the society a specimen, with the following remarks: "I hardly know whether you would call this a pathological specimen or not. But you see a mass which was removed from a uterus, and it proves to be a sponge, A short time ago I was called to see a lady who had been treated by a physician who introduced a sponge tent into the cervix uteri, and instructed the lady to allow it to remain two or three days, and then pull it out. She attempted to do so, but the string broke and the sponge was not obtained. After three weeks of suffering, with a discharge per vaginam, I was called to see her. By digital examination I could not find any evidence of the sponge, the external os being closed so as to merely admit a probe. But I could not find any sponge by probing, so I introduced an Ellinger dilator, and soon seized the sponge with a forceps and brought it out. The symptoms present passed rapidly away, and the patient is now well. The point to be learned is, that when a physician introduces a sponge tent, he himself should remove it.

The society then adjourned.

